# Mathematical modelling and numerical simulation of the morphological development of neurons

**DOI:** 10.1186/1471-2202-7-S1-S9

**Published:** 2006-10-30

**Authors:** Bruce P Graham, Arjen van Ooyen

**Affiliations:** 1 Department of Computing Science and Mathematics, University of Stirling, Stirling FK9 4LA, UK; 2 Department of Experimental Neurophysiology, Vrije Universiteit, De Boelelaan 1085, 1081 HV Amsterdam, The Netherlands

## Abstract

**Background:**

The morphological development of neurons is a very complex process involving both genetic and environmental components. Mathematical modelling and numerical simulation are valuable tools in helping us unravel particular aspects of how individual neurons grow their characteristic morphologies and eventually form appropriate networks with each other.

**Methods:**

A variety of mathematical models that consider (1) neurite initiation (2) neurite elongation (3) axon pathfinding, and (4) neurite branching and dendritic shape formation are reviewed. The different mathematical techniques employed are also described.

**Results:**

Some comparison of modelling results with experimental data is made. A critique of different modelling techniques is given, leading to a proposal for a unified modelling environment for models of neuronal development.

**Conclusion:**

A unified mathematical and numerical simulation framework should lead to an expansion of work on models of neuronal development, as has occurred with compartmental models of neuronal electrical activity.

## Background

A highly distinctive feature of neurons is their morphology. Neurons exhibit long processes, or neurites, that are fundamental to the formation of the connected networks of neurons that constitute a nervous system. One neurite, the axon, forms the output electrical signalling pathway of a neuron. A typical axon has a main trunk from which shorter side branches, or collaterals, emerge to form points of contact with appropriate target neurons. Axons may be extremely long, up to about one metre in humans, for example. The remaining neurites of a neuron are dendrites, which form complex tree-like structures and are the recipients of most synaptic contacts from the axons of other neurons. Different types of neuron can be distinguished by the structure of their dendrites, which can be characterized in terms of segment lengths and diameters, the number of terminals (unbranched tips), the number of branch points, and topological factors such as symmetry [1-3].

Much of the research in the field of computational neuroscience has been directed at understanding the electrical signalling properties of neurons, with a particular emphasis on the impact of complex neuronal morphology on signal integration (e.g. [4-7]). This uses so-called "compartmental" models of neurons [8] which are based upon solving a well understood partial differential equation (PDE) for membrane voltage over space and time, where space is one dimensional along the length of neurites. Powerful numerical techniques for integrating this equation and for coping with the branched structures of neurons have been developed [9,10]. Sophisticated computer simulation packages, such as NEURON [11] and GENESIS [12], make the creation of electrical signalling models a near user-friendly experience. Such packages provide graphical interfaces for both model construction and for the display of results.

However, this research into electrical signalling ignores the fascinating problem of how a neuron's complex morphology is created during nervous system development. Mathematical modelling and numerical simulation are invaluable tools to help us unravel the processes underlying morphological development. The sorts of model that have been investigated vary widely and are typically aimed at a particular aspect, such as target finding by axons, rather than describing the entire development of a neuron. Consequently, no uniform mathematical or numerical techniques have yet emerged to lead to the building of the sort of user-friendly software available for modelling electrical signalling. In this review we consider a range of modelling endeavours exploring aspects of neuronal morphological development. In conclusion we suggest the features of a computer simulation package that could ease the pain of creating new models of neuronal development and eventually allow the exploration of whole neuron development.

## Methods

### Overview

This review is largely about methods for modelling neuronal development, rather than the latest research results. We will, however, point out the major findings of various models along the way. The review is not exhaustive of all theoretical work on neuronal development (for a comprehensive overview see [13]), but concerns mathematical models aimed at understanding the biophysics of the outgrowth of neurites. Most models consider only specific aspects of neurite outgrowth, and so in turn we will look at models of (1) neurite initiation (2) neurite elongation (3) axon pathfinding, and (4) neurite branching and dendritic shape formation.

### Neurite initiation and differentiation

**Figure 1:**
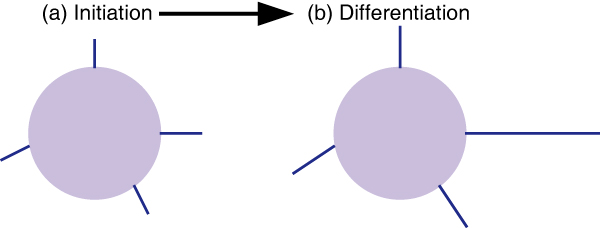
**Neurite initiation and differentiation**. (a) Cell surface inhomogeneities cause instabilities that lead to the formation of broad lamellipodia that condense into short neurites. (b) Competition for a growth-permitting chemical produced in the cell body can lead to the fastest growing neurite becoming the only outgrowing neurite, characteristic of axonal differentiation.

The first problem concerns how neurites form from an initially spherical neuronal cell. Then, subsequent to initiation, there is a process of differentiation in which one of the neurites becomes the axon, and the remaining neurites form the dendrites. These stages of growth are illustrated in Figure 1. Mathematical modelling of these processes has been undertaken by Hentschel and colleagues over the last decade [14-18]. They investigated how initial neurite outgrowth could be established by a positive feedback mechanism triggered by small inhomogeneities in the cell surface [14,15,18]. Calcium is assumed to be the morphogen that promotes cell outgrowth. In an excitable membrane model, local membrane potential is modulated by the influx of sodium through voltage-dependent sodium channels. Calcium influx also occurrs through voltage-dependent channels. Small bulges in the cell surface produce focal depolarizations leading to elevated sodium and calcium entry and hightened calcium-dependent outgrowth of the bulge, eventually leading to neurite formation. Sodium, calcium and voltage gradients are established from the distal to proximal ends of the neurite, in accord with experimental evidence.

The model was simulated using a cellular automata approach in which the cell interior is divided into 1 *μm*^2 ^blocks and the cell surface is described by a linked list of 1 *μm *segments of membrane. Each segment contains information about the local transmembrane potential, the conductance of sodium and calcium and their submembrane concentrations. The concentrations of sodium and calcium in each cytoplasmic block are calculated on the basis of a quasi-equilibrium assumption. Growth proceeds by the addition or subtraction of segments from the cell surface. Full details of the simulation procedure are given in [18]. Further outgrowth of neurites is assumed to be rate-limited by the availability of a particular, but unspecified chemical at each neurite tip [16-18]. This chemical is produced in the cell body and transported by diffusion and active transport to the end of each neurite emanating from the cell body. This scenario was modelled by a set of coupled ordinary differential equations (ODEs) describing the chemical concentration in the cell body, *c*_0_, and in each of *n *neurite terminals, *c*_1 _to *c*_*n*_, and the lengths of each neurite, *l*_1 _to *l*_*n*_. The equations are:



where *T*_*i *_is the transfer rate of chemical from the soma to a neurite tip, *D *is the diffusion constant, *A *is the cross-sectional area of a neurite, *V*_0 _is the volume of the soma, *V*_*i *_is the volume of a neurite tip, and *F *is a growth-dependent active transport rate. The elongation rate of a neurite is proportional (*α*) to the concentration in the tip, *c*_*i*_, and chemical is consumed in the tip at a rate proportional (*G*) to the elongation rate.

A "winner-take-all" dynamic instability can emerge if one neurite has a slightly larger initial growth rate. This leads to greater consumption of the chemical at this neurite tip and the subsequent capturing of more chemical by this neurite than by the others. This quickly leads to rapid growth by this one neurite, with all other neurites having only very slow growth. This is characteristic of the differential growth of the neurite that becomes the axon in real neurons. This model is able to reproduce the results of a number of axotomy experiments in which severing of the nascent axon at different lengths, relative to the other neurites, results in either the axon reestablishing itself or another neurite becoming the new axon [18]. Such competitive effects between neurites have also been investigated in similar ODE models of the growth of branched dendritic structures [19-21].

### Neurite elongation

**Figure 2:**
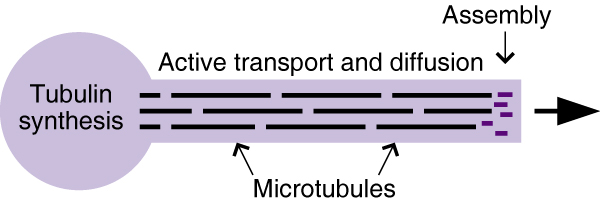
**Neurite elongation**. Construction of the microtubule cytoskeleton is a rate-limiting factor in neurite elongation. The rate of construction is determined by the production and transport of tubulin along a neurite, and the assembly of tubulin onto the ends of microtubules at the neurite tip.

Following initiation, neurites elongate and branch, reaching lengths of tens to hundreds of micrometres for dendrites, to over a metre or more for long axons. Modelling has been used to explore just how neurites elongate and the biophysical constraints on the lengths that may be achieved. This work has centred around the dynamics of cytoskeleton construction, particularly of the microtubules that form the major supporting scaffold within neurites [19,22-24]. Elongation is assumed to be a function of the amount of available free tubulin at the growing tip of a neurite, which is assembled onto the ends of microtubules, extending the internal cytoskeleton. The constraints to growth are the production rate of tubulin and its transport from its site of synthesis, typically assumed to be the cell body (but see Alvarez et al [25]), to the neurite tip by diffusion and active transport. An illustration of this model is given in Figure 2. The most mathematically sophisticated treatment of this problem is the continuum model developed by McLean and colleagues [23,24] in which a PDE describes the tubulin concentration along the length of a single, unbranched neurite. This model essentially consists of the following four equations:



The free tubulin concentration at a point *x *in the neurite at time *t *is given by *c*(*x*, *t*). The length of the neurite at time *t *is given by *l*(*t*). Space *x *lies in the domain 0 to *l*. Tubulin moves by active transport (*a*) and diffusion (*D*), and degrades with rate *g*. Synthesis of tubulin in the cell body at rate *∈*_0_*c*_0 _results in a flux of tubulin across the boundary at *x *= 0. Assembly of tubulin onto microtubules results in a flux of tubulin across the boundary at *x = l *(assembly flux *∈*_*l *_and return flux *ζ*_*l*_) and a change in length (assembly rate *r*_*g *_and disassembly *s*_*g*_).

This model is a generalisation of previous ODE and algebraic treatments of this scenario [19,22]. Its solution, both analytically and numerically, is complicated by the fact that it is of the moving boundary type ie the spatial domain changes over time due to changes in the length, *l*. A stable and accurate numerical solution has been proposed in which the spatial domain is discretized into a fixed number of *N *grid points and integration is carried out on a spatially-transformed domain in which *x *lies only in 0 to 1 [26]. Transforming back into the real length domain results in the grid points growing further apart as the length increases.

Steady-state analysis has revealed that growth can proceed in three different regimes, as determined by the relative proportions of *construction*, due to the synthesis and active transport of tubulin and its assembly onto microtubules, and *dissipation*, due to tubulin diffusion, degradation and its disassembly from microtubules [23,24]. If construction dominates dissipation, then large growth results. If dissipation dominates construction then only short lengths are achieved. Moderate growth ensues if construction and dissipation are approximately balanced. The transition between growth regimes is highly nonlinear. Growth in the large and short regimes is quite damped and is determined by active transport in the large regime and diffusion in the short regime. Oscillations in length can occur in the moderate regime where growth is affected by both the active transport and diffusion of tubulin [26]. The ODE models described so far only prescribe concentrations at a small number of points, typically the cell body and neurite tips (but see work on compartmental models, described below). Numerical solution of this PDE model enables the visualisation of tubulin concentration over space and time, to any desired resolution, as the neurite grows [23].

Aspects of this model have been the subject of more detailed treatments. Active transport of tubulin is here assumed to proceed with a flux proportional to the local concentration. In reality, tubulin dimers undergo periods of active transport when they are attached to the molecular motors, interspersed with periods of free diffusion. A PDE reaction-diffusion-transport model that covers this scenario, and considers both uni- and bidirectional motors for the active transport of intracellular organelles has been investigated by Smith and Simmons [27].

Modelling has also been directed at examining the detailed process of microtubule assembly. Monte Carlo simulation of the reaction and diffusion of individual tubulin dimers near the tip of a microtubule has been used to investigate the outgrowth dynamics of a microtubule and the sources of variability that might underly the observed dynamic instability in which a microtubule may switch from elongation to retraction and vice versa [28]. A mean field approach has been used to determine the diffusion-limited rate of microtubule assembly, with the conclusion that observed assembly rates are not limited by the diffusion of free tubulin [29].

The growth cone at the tip of a neurite also plays a significant part in neurite elongation and is not explicitly included in the microtubule assembly models. However, microtubules extend into the growth cone and interactions between the growth cone and microtubules are significant for neurite outgrowth. Microtubules exhibit so-called dynamic instability, the random alternation between microtubule growth and shrinkage. This process plays an important role in the motility of growth cones and their finger-like protrusions, the filopodia. The volume of a growth cone is so small that growing or shrinking microtubules are expected to cause fluctuations in the concentration of free tubulin. Monte Carlo simulations have shown that these fluctuations have a significant effect on microtubule dynamic instability [30]. They shorten microtubule growth and shrinkage times and change their distributions from exponential to non-exponential, gamma-like, which, interestingly, allow optimal search behaviour by microtubules [31]. Thus, growth cone volume, via its influence on the fluctuations in the concentration of free tubulin, can affect dynamic instability and, consequently, growth cone motility.

The growth cone exerts tension on the trailing neurite which influences microtubule assembly rates. Precisely how tension affects assembly has been the subject of a number of modelling studies (reviewed in [32]). Physical considerations essentially lead to a description in which the rate of microtubule assembly is an inverse function of the elastic tension, *F*, on the microtubules due to stretching of the neurite:



where *l *is microtubule length, *r*_*g *_is the unmodified assembly rate and *s*_*g *_is the disassembly rate (assumed not to be a affected by tension). Tension from the growth cone can relieve this elastic tension (lowering *F*) and thus promote assembly and outgrowth. In the PDE model described above, all tensions are assumed to be constant throughout. A description that considers variation of tension over time needs the inclusion of a hetergeneous environment that is detected by the advancing growth cone. Such models have been formulated for studying axon guidance, as described below.

The growth cone itself contains a complex actin cytoskeleton. It has been proposed that this cytoskeleton, when supporting lamellipodia at the leading edge of the cone, undergoes a caterpillar like movement to propel the growth cone along [33]. A more detailed modelling study of actin cytoskeleton dynamics in lamellipodia supporting cell movement is reported in [34].

### Axon pathfinding

A fundamental feature of the outgrowth of an axon is its ability to follow a path to an appropriate target. This requires the axon's growth cone to sense environmental cues and to be able to turn towards the desired direction, or away from an incorrect location. Modelling efforts have been directed at the growth cone's ability to sense external chemical gradients [35], its turning ability [32,33,36], and the formation of nerve tracts in which groups of related axons bundle together and travel to a remote target region [37,38]. Related work, which will not be covered here, includes the problem of topographic map formation in which the topography of the sending region of neurons is preserved in the pattern of connections formed by the arriving axons over the target region [39].

The most fully worked example of axonal elongation and direction finding is that of Aeschlimann [32,36].

**Figure 3:**
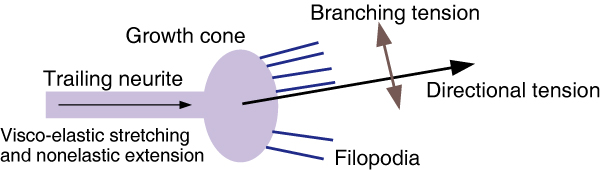
**Axonal pathfinding**. Detection of chemoattractants in the external environment by filopodia produces tension on the growth cone in particular directions. The growth cone will turn towards and grow along the dominant direction. If similar forces are exerted on opposite sides of the cone, the tension may be enough to split the cone into two, leading to the formation of daughter branches.

She considers the outgrowth of a single, unbranched axon in a two-dimensional external environment containing chemical gradients and glial cells (Figure 3). Elongation and bending of the axon is determined by visco-elastic stretching and nonelastic extension due to microtubule assembly. Direction finding and steering is the function of filopodia extending from a circular growth cone at the axon tip. Filopodia elongate to a fixed maximum length, then retract. Retracting filopodia exert tension on the growth cone, which in turn influences axon elongation. The direction of elongation is determined by the vector of forces generated by the distribution of filopodia on the surface of the growth cone. The probability that a new filopodium will emerge at a point on the surface is determined by the local submembrane calcium concentration, which is influenced by existing filopodia. A filopodium can detect a chemical gradient along its length [35] or physical contact with an external glial cell, with both signals resulting in an influx of calcium into the growth cone at the base of the filopodium. This results in a positive feedback, with the calcium concentration being increased most around existing filopodia sensing the largest gradients, and in turn increasing the probability of new filopodia emerging in this area. Localised groups of filopodia contribute strongly to the tension vector and can thus cause the growth cone to turn towards and grow along the maximum external concentration gradient of an attracting chemical. This complex model contains many components and includes a coupled ODE description of neurite elongation and probabilistic descriptions of filopodium formation.

Maskery et al [40] use a much simpler model of axonal pathfinding in which a growth cone undergoes stochastic changes in direction or is repulsed by external cues to demonstrate that guidance by external cues is most effective when growth is at a transition between being dominated by stochastic or deterministic effects.

Models of how groups of growing axons form bundles, or fascicles, have been explored by Hentschel and van Ooyen [37]. In their most efficient (de)bundling model, a diffusible signal released by the target region provides long-range axon guidance. Bundling of the axons occurs due to a diffusible chemoattractant released by the axonal growth cones, causing the axons to grow towards each other. Debundling of the axons as they come near the target occurs due to a chemorepellant released by the axonal growth cones, with the amount of chemorepellant being a function of the concentration of the target-released attractant. Debundling must occur for the axons to spread out and innervate the full extent of the target region. The chemoattractant and repellant released by the axonal growth cones remain hypothetical. They could plausibly be mediated by contact interactions between growth cone filopodia, rather than be diffusible molecules [37].

The two dimensional implementation of this scenario involves treating each target cell and each growth cone as a point source for attractant or repellant. The quasi-steady-state concentration gradient of each chemical from each source is calculated and summed. The change in position, **r**_*α*_, of each growth cone, *α*, is then calculated as a function of the two attractive and one repulsive gradients:



where ∇*ρ*_*x *_is the concentration gradient of growth cone (*c*) or target (*t*) attractant, or growth cone repellant (*r*) and *λ*_*x *_is the associated growth rate constant.

Segev and Ben-Jacob [41,42] have used a similar modelling approach to explore more general chemotactic effects in neuronal network formation in a 2D environment. In their models single axons grow out from each soma, with their direction of growth being determined by a variety of attractive and repulsive environmental cues. In particular, each soma is assumed to emit a repulsive signal. Once an axon reaches a specified length it emits a pulse of an attractive signal. A soma then responds by itself emitting an attractive signal if it detects an above-threshold concentration of growth cone attractor. As a consequence a growth cone is then attracted to the nearest soma. This model was used to investigate the patterns of network connectivity that could result from various initial spatial distributions of somas. In some simulations spatial distributions of glial cells that emitted chemotactic signals were also present. Validation of these models awaits new measures for characterizing and distinguishing between different network organisations.

### Neurite branching and dendritic shape formation

Neurites do not just elongate, they also make repeated branches. Branch formation is either due to a bifurcation of the growth cone, or the interstitial formation of a new branch part way along an existing neurite. Growth cone bifurcation seems to largely underly the formation of many different types of dendrite [43]. Interstitial branching is important for the formation of axon collaterals [43] and also for the creation of short oblique branches in certain dendrites [44]. We will concentrate here on mathematical models that consider the biophysics of growth cone bifurcation and the implications for the creation of the dendritic structures that characterize different neuronal types. The models can be divided into those that describe external influences on the growth cone and those that consider internal constraints on cytoskeletal construction and stability. There is also a range of statistical models that are aimed at the reconstruction of realistic dendrites, without necessarily describing the developmental process (see for example [45,46]). Such models will not be considered here.

Models of branching due to external influences consider tension on the growth cone due to filopodia sensing cues in different directions in the environment [47,48]. If there is sufficient tension in opposing directions on different sides of the growth cone, then the cone splits apart to form two daughter branches, which proceed to elongate in the direction of the sensed cues (Figure 3). These models consider growth along a plane, equivalent to an isolated neuron growing in culture. The external environment is populated by attracting cues, which are either point sources or continuous chemical gradients. A number of filopodia radiate from a point growth cone, with direction and tension dependent on the pattern of external cues. A tension threshold determines when the growth cone branches as it elongates through the environment. The micro-details of the internal actin cytoskeleton, its stability and how it is rearranged following a branching event are not considered in these simple models. The output of these models is comparable to data on branching angles and segment lengths from cells grown in tissue culture [49].

Theoretical studies indicate that dendritic branching angles may follow a principle of neurite volume minimisation [50], or equivalently, equilibration of tension forces on growth cones [51]. Network formation may refine branch angles to minimise neurite lengths [52]. Statistical analysis also indicates a strong tendency for neurites to grow away from their parent cell body, thus influencing branching angles [53]. Models of internal constraints are similar to the models of elongation based on the construction of the microtubule cytoskeleton. In addition to considering the rate of neurite elongation due to microtubule assembly, these models also consider the stability of the resultant microtubule bundles as determining the likelihood for a neurite tip to bifurcate. As with the external models, an explicit model of the growth cone and its actin cytoskeleton currently are not included.

The basic model considers whether the production and transport of an unspecified branch-determining substance imparts constraints on branching [54]. Statistical models of dendrite formation indicate that the probability of a neurite bifurcating is modulated as the tree grows and is a function of the number of terminals in the tree and the number of branch points between a particular terminal and the cell body (centrifugal order) [3]. The basic model shows that modulation of branching probability as a function of the number of terminals and their centrifugal order can arise from variations in the availability of a branch-determining substance at each terminal due to the diffusion and active transport of that substance from its site of production in the cell body. Such a substance can plausibly be identified as tubulin, or possibly a microtubule-associated protein, such as MAP2, that influences the stability of microtubule bundles in dendrites.

**Figure 4:**
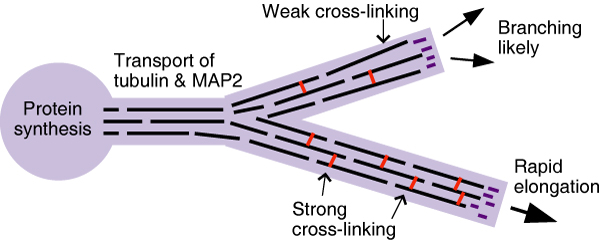
**Neurite branching**. Construction and stability of the microtubule cytoskeleton may determine both elongation and branching rates. Microtubule bundles are cross-linked by microtubule-associated proteins, such as MAP2. Dephosphorylated MAP2 stabilises the bundles and promotes microtubule assembly, and hence elongation. Phosphorylated MAP2 loses its cross-linking ability, destabilising the bundles and thus increasing the likelihood of neurite branching.

More sophisticated models describe both elongation and the branching rate explicitly as functions of tubulin and MAP2 concentrations at a terminal (Figure 4) [55,56]. Both tubulin and MAP2 are assumed to be synthesised in the cell body and transported by diffusion and active transport along the growing dendrites. At a terminal, the amount of free tubulin and MAP2, and the phosphorlyation state of MAP2 determine the elongation and branching rates. Dephosphorylated MAP2 acts as a cross-linker between microtubules, stabilising the bundles and promoting microtubule assembly. Phosphorylated MAP2, on the other hand, loses its cross-linking ability and destabilises the microtubule bundles, thus increasing the likelihood of a bifurcation event. (De)phosphorylation of MAP2 is a Michaelis-Menten function of the calcium concentration, with small changes in calcium possibly resulting in a large shift in the balance between phosphorylated and dephosphorylated MAP2. Calcium entry is assumed along the length of the dendrite (putatively through voltage-gated channels). The biochemical (de)phosphorylation pathways, involving at least CaMKII and calcineurin, are not explicitly modelled. The elongation and branching rates are given by:



where *c*_*l *_is the concentration of free tubulin at a neurite tip, *B*_*l *_is the concentration of dephosphorylated and microtubule-bound MAP2, *P*_*l *_is the concentration of phosphorylated MAP2 and *P*_*b*_, is the probability of branching.

This complex model of elongation and branching has been implemented as a coupled system of ODEs, with their numerical solution following a 'compartmental" approach, as has been used for solving the voltage equation for simulating electrical activity. In this approach, a neurite is divided into a number of short compartments, with chemical concentrations being calculated for the volume of each compartment. Chemicals move between compartments due to bulk diffusion and active transport. As a neurite elongates, new compartments are added when needed. New compartments are also created when a branching event occurs. Various strategies for when and where new compartments are added have been investigated [57]. As with PDE models, this approach also allows visualisation of concentration gradients along the length of neurites, in contrast to ODE models that only consider concentrations in the cell body and in neurite terminals [19,20,37,54]. Work is needed to reconcile this compartmental approach with the numerical solution of the PDE model of neurite elongation [23,24,26].

## Results and Discussion

### Modelling results and experimental data

All of the models described here are aimed at understanding the biophysics of various processes underlying the morphological development of a neuron. They seek to support explanations for the rate and direction of outgrowth, segment lengths, branching angles and the branching structure of dendrites and axons. Different models have been successful at matching experimental data for some but not all of these features at the same time.

Much of the work draws its motivation from statistical models of neurite outgrowth, with the aim of providing biophysical explanations for statistical phenomena. Dendrites exhibit correlations between segment diameters, lengths and segment branching probabilities [1,2,44]. Complex models of neurite elongation and branching [55,56] predict variations in calcium concentration over time at neurite tips that could alter the balance between elongation and branching through effecting the phosphorylation state of MAP2. These models can produce the trends in segment lengths and branching formation seen in real dendrites. Statistical branching probabilities seem to be a function of the size and topology of the growing dendrite [3] and it has been demonstrated that such a dependency could arise through constraints on the transport of growth determining substances, such as tubulin, throughout the growing tree [54,58]. Tight matches to experimentally measured dendritic topologies have been achieved with this model. Limitations on intracellular transport are also predicted to result in competition for outgrowth between neurites [16,19,20]. An aspect that remains to be captured in biophysically-based models is how the diameter of a neurite segment evolves.

Predictions have been made about the requirements and limitations of extracellular signals for axon guidance and bundling [35,37,40-42]. Temporal and spatial requirements for attractive and repulsive cues to achieve appropriate target finding are suggested, but remain to be confirmed experimentally. For example, debundling may require axonal growth cones to release a repellant as they near their target region [37]. Release of an attractant by a growth cone in a target region that in turn triggers attractant release by target cells may be required for appropriate network connection formation [41,42]. A particular feature of a number of the models is the role of calcium as a second messenger and the importance of spatial inhomogeneities in calcium concentration within a cell. This includes submembrane calcium hotspots in the cell body driving neurite initiation [14,15], submembrane calcium hotspots at the tip of a growth cone influencing filopodium initiation and ultimately growth direction [32], and changes in calcium concentration at a neurite tip altering the phosphorylation state of MAP2 and ultimately the balance between elongation and branching [55,56]. Calcium imaging is beginning to provide data to confirm or deny these models and to help generate new models [18]. Also, no explicit description is attempted in these models of the precise biochemical pathways which lead from calcium to the end effect. Detailed second messenger pathway modelling is a feature of systems biology and sufficient data is available to attempt more detailed modelling here [59].

In summary, biophysically-based models are making predictions about the nature of intracellular and extracellular constraints that could determine the neuronal morphologies and network topologies seen experimentally. Such models consider the dynamics of cytoskeleton construction, the effect of tension on neurite outgrowth, and the temporal and spatial requirements for chemotactic signals.

### Modelling techniques

A major feature of models of neuronal development is the necessity to calculate quantities that vary over space as well as time, with the significant complication that the spatial domain itself varies with time. The natural mathematical specification of such problems is the partial differential equation (PDE). The formulation and numerical solution of PDE models for which the spatial domain changes with time are of the moving boundary type and are more difficult to deal with than models with a fixed spatial domain. Nonetheless a PDE model for neurite elongation has been developed [24], but awaits extension to cover a fully branched neuritic structure. In this model, space is one dimensional along the interior of the neurite. Full details of the numerical solution of this model are given in [26].

A two dimensional PDE model of an external environment of arbitrary geometry and containing various point sources of chemical gradients has been formulated and solved numerically [38]. The moving tips of growing neurites have a spatial location within this environment, but the neurite itself has no spatial extent. This framework can be used to model axon guidance in response to both diffusible and non-diffusible, membrane bound molecules. It also allows for the (hypothetical) possibility that growth cones secrete diffusible molecules upon which they respond themselves. The internal state and geometry of the growth cone, which may affect how the growth cone responds to chemical gradients, can also be incorporated. Thus, a wide range of axon guidance models, including those involved in topographic mapping, can be implemented and explored. The framework could easily be extended to include neurite branching due to external influences.

While the mathematical rigour provided by the PDE approach is highly desirable, the flexibility of a "compartmental" approach using a system of coupled ODEs is also potentially very useful. This is particularly evident in simulation software for modelling electrical activity in neurons (e.g. NEURON and GENESIS) in which nonlinear models of ion channel activity are easily incorporated and coupled to the underlying membrane voltage equation. Spatially inhomogeneous distributions of ion channels are easily handled, as are "point processes", such as synapses, that only occur at particular locations. The natural extension of this approach to the scenario of a neuron undergoing morphological development is the addition or deletion of new membrane "compartments" over time [57]. This means that new points are added (or deleted) to the spatial grid on which calculations are made, and existing points are fixed in space. This contrasts to the spatial grid used to numerically solve the PDE model of neurite elongation [26] in which the grid contains a fixed number of points but the mesh is stretched in space to account for growth, effectively moving each point in space over time. Fixing as many points as possible in space is useful when modelling the interaction of an outgrowing neurite with its environment, which may contain chemical gradients, fixed chemical markers and other growing neurites, for example. Exact spatial relationships between objects then are more easily maintained, such as the contact point of a synapse on a neurite. The "compartmental" approach can also allow for the easy incorporation of new ODE, algebraic or probabilistic equations describing new intra- or extracellular components in a spatially inhomogeneous way.

### Towards a unified modelling environment for neuronal development

From both a conceptual and a mathematical point of view it is possible to envisage a unified scenario that encompasses many of the individual models described above. Most of the models lie within the framework of a two-dimensional external environment coupled with a one-dimensional intracellular space. The external environment contains either point sources or continuous chemical gradients of attractors and repellors. Physical barriers, such as other cells, could also be present. The intracellular space describes the concentration of various chemicals along the length of neurites as determined by their sites of production and their transport by diffusion or active motors. Exceptions to this description are the two-dimensional intracellular space used to model neurite initiation [15] and growth cone locomotion [33]. A description that would combine these features would consist of a neuronal model with one spatial dimension along the intracellular length of neurites, possibly with two dimensional descriptions of the cell body and growth cones. The neuronal model would interact with a two dimensional description of the extracellular environment which may contain chemical gradients and other cells that could be detected by and influence the development of a neuron. The intracellular quantities of interest are the spatial concentration profiles of mo lecules involved in neurite outgrowth, such as tubulin. Sites of synthesis or influx and the one dimensional diffusion and active transport of molecules must be specified. Reactions between molecules may take place at predefined points or along the length of the neurite. Sources of attracting or repelling chemical factors and their static, or quasi-static gradients must be specified in the external environment. Growing neurons and other cells, such as glia, must occupy space in the external environment, thus allowing physical contact between cells.

Appropriate numerical schemes for implementing the required 2D external environment [38,41] and the ID intracellular environment [26,57] are available. Extending these schemes to include a 3D external environment should be conceptually straightforward, but would increase the computational burden considerably. The state vector approach employed by [38] to describe the interaction between growth cones and the external environment provides the framework required for combining complex models of cytoskeleton construction [55,56] with growth in an environment. It will, however, be necessary to account for interaction between a neurite and the environment at any point along the neurite, not just at the growth cone. Extra complexity also arises if it is necessary to take account of the space-filing of a developing neuron. This is certainly required to accurately describe growth in a multicellular environment. In this case both the internal and external spatial grids must be adaptable or deformable. The typical time scale for neuronal growth driven by chemical concentrations is on the order of minutes to hours. Growth is also known to be affected by electrical activity and synapse formation. These effects are only implicit in the models described here [18,55,56]. Explicit modelling of electrical activity requires a time scale of micro- or at most milli-seconds. It is not feasible to contemplate modelling neuronal development, which may proceed for days, on this time scale. So the inclusion in a model of the effect of explicit electrical activity on the developmental process would require an adaptive approach in which the model switches from development to modelling short periods of electrical activity. A calculation of the average effects of electrical activity could then be carried forward into the next period of developmental modelling.

Thus an appropriate unified modelling environment needs to encompass both varying spatial and temporal scales and inhomogeneities in the components of a given model. Appropriate and diverse mathematical techniques are required for calculating quantities at different scales of interest. These techniques are available individually, but a numerical simulation package that incorporates all required techniques to implement the unified modelling environment outlined here remains to be built.

## Conclusion

Mathematical modelling and numerical simulation are powerful tools to help us understand neuroscientific experimental data and ultimately the operation of the nervous system. User-friendly simulation software has led to an enormous body of work on modelling electrical activity in morphologically complex neurons and networks of neurons. Mathematical models of aspects of nervous system development are more disparate in nature but there is some commonality of themes and mathematical techniques, as reviewed here. Powerful simulation environments are beginning to be developed and this will hopefully lead to an expansion in the use of mathematical modelling to understand development of the nervous system.
